# Case Analysis and Literature Review of Thirteen Patients with Autoimmune Encephalitis

**DOI:** 10.1155/2022/4802480

**Published:** 2022-01-25

**Authors:** Anqi Huang, Jingru Wang, De-peng Feng, Weiwei Wang, Xueli Li

**Affiliations:** ^1^Department of Neurology, Liaocheng People's Hospital and Liaocheng School of Clinical Medicine, Shandong First Medical University, Liaocheng, Shandong 252000, China; ^2^Department of Neuroimmune Laboratory, Liaocheng People's Hospital and Liaocheng School of Clinical Medicine, Shandong First Medical University, Liaocheng, Shandong 252000, China

## Abstract

**Objective:**

To investigate the clinical manifestations, laboratory and imaging examinations, and the treatment outcomes of autoimmune encephalitis (AE).

**Methods:**

The clinical data of 13 patients with autoimmune encephalitis who were hospitalized in the department of neurology, Liaocheng People's Hospital from July 2016 to August 2018 were retrospectively analyzed.

**Results:**

The average age of onset of the 13 patients was 45 years, including 6 cases (46%) of anti-NMDAR encephalitis, 3 cases (23%) of anti-GABAB receptor encephalitis, and 4 cases (30%) of anti-LG11 encephalitis, and 4 of them showed abnormal signals of brain MRI (30%). 13 patients (100%) had cognitive impairment and psychiatric symptoms; seizures occurred in 12 patients (92%); lung cancer was found in 1 patient (7%). One case was given up because of the treatment of lung cancer, the other was controlled obviously in epilepsy, and cognitive impairment and abnormal mental behavior were also significantly improved.

**Conclusion:**

Patients with AE still need to be diagnosed early to avoid missed diagnosis and receive early immunosuppressive therapy, which could effectively reduce mortality and morbidity. A detailed history, clinical manifestations, and positive results for specific NSAbs tests can confirm the diagnosis, and the treatment is mainly done by immunosuppressive therapy.

## 1. Introduction

Autoimmune encephalitis (AE) is a serious disease characterized by seizures, mental disorders, and clinical cognitive decline of the central nervous system caused by the autoimmune reaction in inflammatory diseases [1]. In 2007, Dalmau et al. found N-methyl-D-aspartate (NMDA) antibodies in the serum and cerebrospinal fluid (CSF) of 12 female patients with teratoma [2]. The development in the research of neuroimmunology and the continuous discovery of new autoantibodies have gradually deepened the understanding of AE [3]. However, there is a lack of a unified AE diagnostic standard in the world at present. An early diagnosis of AE is very difficult, and it can be easily confused with infectious encephalitis. It has been reported that immunosuppressive therapy has a significant positive effect in most cases of AE, which could make the prognosis of AE is better than other types of encephalitis. Therefore, the early diagnosis and treatment of immunosuppressive therapy are conducive for alleviating the condition, restoring health, and reducing mortality and morbidity. In this study, a detailed analysis is performed on 13 cases of patients with AE in the neurology department of the hospital in recent years. The data of their age, gender, clinical manifestations, brain MRI, EEG, CSF test, related blood tests, treatment, and efficacy were analyzed, which may help to improve the awareness of the neurological physician about the disease to achieve early diagnosis and early treatment.

## 2. Methods and Materials

### 2.1. Subjects

A total of 13 patients were diagnosed with AE in our hospital from July 2016 to August 2018, including 9 males and 4 females, aged from 15 to 68 years with an average age of 45 years.

### 2.2. Methods

All patients were analyzed for clinical manifestations including the age of onset, first symptom, whether there were prodromal symptoms, mental disorders, seizures, cognitive dysfunction, central hypoventilation, symptoms of autonomic dysfunction, and treatment regimens. Additionally, further information was obtained from laboratory tests, magnetic resonance imaging (MRI) of the head, computed tomography (CT) scan of the chest, full-B ultrasound examination (including the liver, gall bladder, pancreas, spleen, kidney, gastrointestinal tract, and genitourinary tract), CSF routine and antibody test, and electroencephalogram (EEG) data, as well as from observation of the treatment and prognosis in the patients. All blood and CSF samples of the patients were detected for AE-related antibodies in our hospital neuroimmunology laboratory.

## 3. Results

In total, 13 patients were diagnosed with AE, including six anti-NMDA receptor encephalitis, three anti-*γ*-aminobutyric acid B (GABAB) receptor encephalitis, and four anti-leucine-rich glioma 1 (LG11) antibody. Nine patients were male, and four were female. The average age of onset was 45 years (from 15 to 68 years).

The onset of the symptoms was either subacute (3 patients) or acute (10 patients). Three cases had a history of flu. Other presentations included the development of seizures (7 patients), psychological symptoms (5 patients), and advanced dementia (1 patient).

All the patients showed different degrees of cognitive dysfunction and psychotic symptoms, of which 6 patients presented unconsciousness, while the others had varying degrees of cognitive decline and mental disorders including advanced hypothyroidism, auditory hallucination, hallucination, delusion, anxiety and depression, personality changes, and irritability.

All the patients experienced different types of seizures during the course of their illness, such as complex partial seizures, myoclonus, generalized tonic-clonic seizures, and status epilepticus. Four patients had intractable epilepsy, and 1 patient had frequent episodes of epilepsy even after the anticonvulsant treatment with drugs such as phenobarbital, valproate, levetiracetam, midazolam, and propofol. Although 2 patients responded to the “phenobarbital, sodium valproate, propofol, and midazolam” quadruple antiepileptic treatment, they still experienced seizure attacks sometimes. Additionally, 2 patients developed involuntary movement of the tongue, face, and limbs, and 1 patient developed symptoms of autonomic dysfunction with partial chills and skin pilus. Four patients developed central hypoventilation and needed assisted mechanical ventilation. During the current follow-up, there was recurrence in 1 patient.

The clinical data of the patients are summarized in Table 1. The routine CSF tests of 8 patients showed that there was an increase in the CSF pressure in 3 patients and cell number in 5 patients (26–58 × 10^6^/L). No quantitative abnormalities were observed in the CSF. One case of anti-GABAB receptor encephalitis showed positive results of oligoclonal bands both in the CSF and in the blood. The results of AE antibody testing in the CSF and blood of all 13 patients are summarized as follows: 6 anti-NMDA antibody-positive patients, 3 anti-GABAB receptor antibody-positive patients, and 4 anti-LG11 antibody-positive patients.

**Figure 1:**
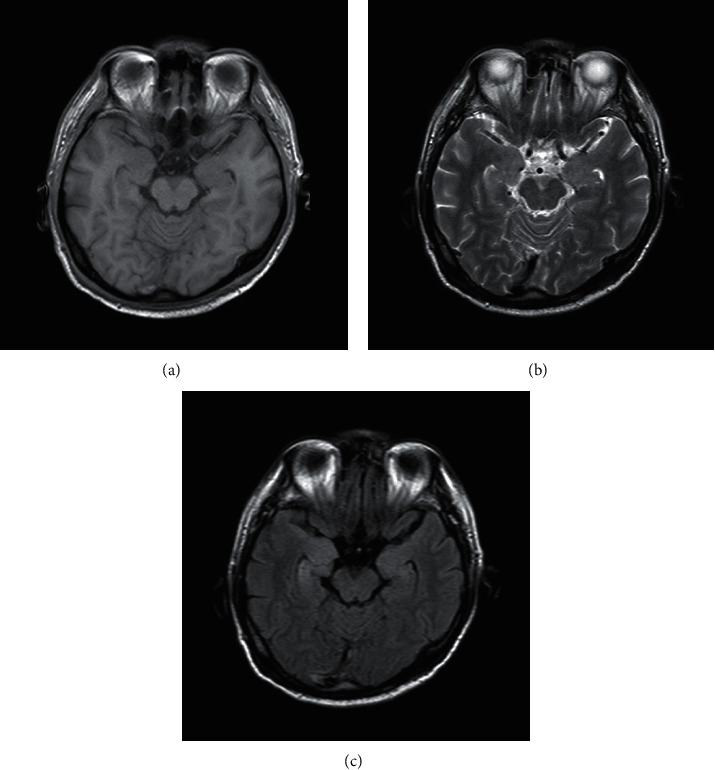
The cranial brain MRI of one case of anti-LG1 receptor encephalitis: (a) bilateral long tabular hippocampal T1, (b) slightly longer signal T2, and (c) FLAIR sequence with a slightly higher signal and repeat brain MRI showed bilateral hippocampal swelling.

**Figure 2:**
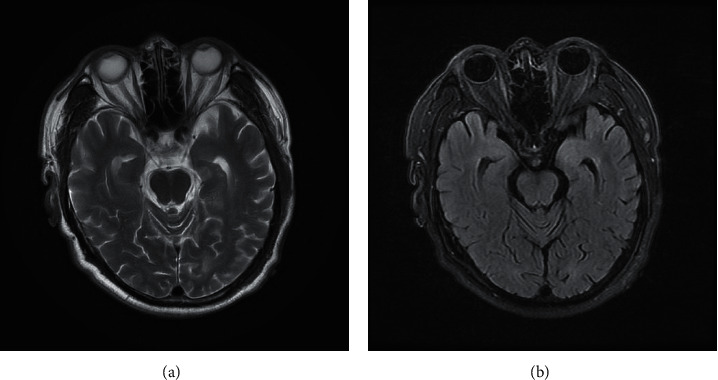
The cranial brain MRI of one case of anti-GABAB receptor encephalitis showed bilateral hippocampal swelling.

**Figure 3:**
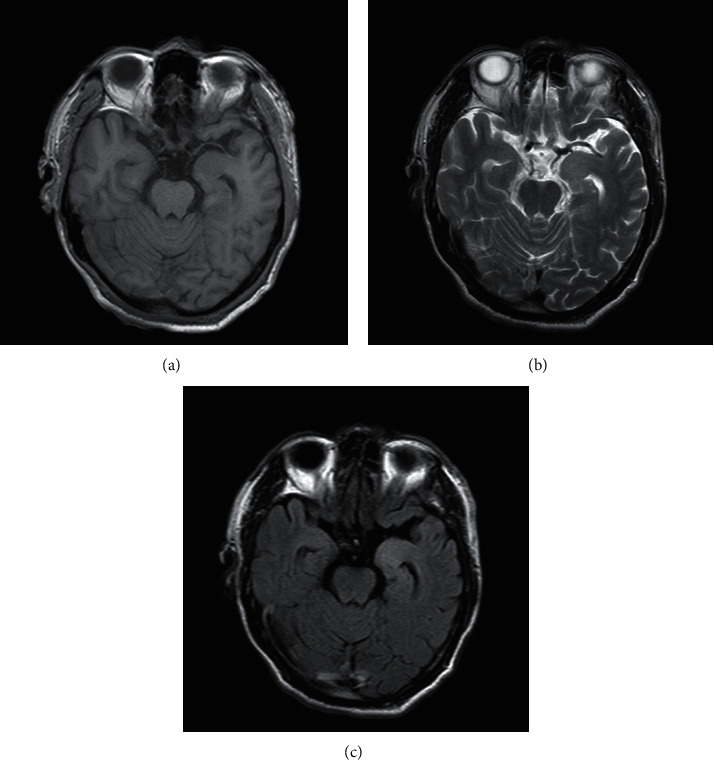
The cranial brain MRI of anti-LG11 antibody-positive encephalitis recurrence in the follow-up: abnormal signal on the left side of the hippocampus, (a) flaky long T1, (b) slightly longer T2 signal, and (c) FLAIR sequence shows a slightly higher signal.

According to the imaging examination, the following results were obtained: 4 patients had abnormal brain MRI associated with the incidence of AE, 2 patients with positive of anti-LG11 showed bilateral long tabular hippocampal T1, slightly longer T2 signal, fluid-attenuated inversion recovery (FLAIR) sequence showed a slightly higher signal, and the repeat brain MRI showed bilateral hippocampal swelling (Figure 1). The brain MRI of the 2 cases of anti-NMDA receptor encephalitis showed multiple intracerebral anomalies with a slightly longer signal of T2, and the FLAIR sequence of the same showed a high signal as well. Diffusion-weighted imaging (DWI) showed bilateral diffusion in the frontotemporal cortex (Figure 2). In addition, another case of anti-LG11 antibody-positive encephalitis recurrence was observed in the current follow-up, and the cranial brain MRI of the same showed abnormal signal on the left side of the hippocampus, flaky long T1, and slightly longer T2 signal with a slightly higher signal in the FLAIR sequence (Figure 3). The remaining imaging examinations showed no correlation with this incidence.

All 13 patients underwent primary tumor screening by abdominal B-scans and chest CT scans. The chest X-ray result of 1 patient with anti-GABAB receptor showed space occupied by the right upper lung without definite pathological examination. The EEG examination of 4 patients with refractory epilepsy showed a widespread diffuse slow wave, and the sharp waves and spikes spread over the whole brain while the wide spike wave was discharged mainly in the frontal and temporal lobes during the attack period. The remaining EEG examination results were roughly normal.

Several treatment strategies were used for the patients. Eight patients were treated with hormones (1 g/d) plus gamma globulin (400 mg/kg), 3 of which were treated with gamma globulin twice in a row, 4 with hormone alone, and none of the thirteen patients were immunosuppressed.

On analyzing the prognosis (6 months) of all the patients, it was found that the neurological function returned to normal in 6 patients. On the other hand, seizures still occurred in 1 patient, and the advanced mental retardation and psychiatric symptoms remained in 4 patients. Additionally, 1 patient had out-of-hospital death due to status epilepticus, and 1 patient suffered involuntary exercise.

## 4. Discussion

Numerous neuronal surface autoantibodies (NSAbs) that are against the surface of nerve cells or synaptic structure of the antibodies have a direct pathogenic role that can disturb the normal function of the cell surface protein. At present, more than 10 NSAbs have been confirmed [4], and anti-NMDAR encephalitis is the most common AE [5].

The patients with anti-NMDAR encephalitis are mostly women with an average age of onset of 21 years. Approximately, 70% of the patients had presymptomatic headaches, fever, vomiting, diarrhea, and upper respiratory tract infections. The typical manifestation is the neuropsychiatric symptoms in the early stage [6, 7], followed by progressive worsening of cognitive dysfunction and confusion [5, 8, 9]. There was the presence of partial or complete seizures during the disease (even status epilepticus), which might be refractory epilepsy [10], and the antiepileptic drugs were generally ineffective but the response improved after receiving immunotherapy. The seizures also manifested as autonomic dysfunction and required intensive care when the patient experienced coma central hypopnea [5, 7, 9].

In this study, out of the 13 patients, 6 had anti-NMDA encephalitis, with an average age of 40 years. There were 2 males and 4 females; 4 patients had a history of cold before the onset of the illness, and 4 patients had psychiatric symptoms. Out of the total cases, 6 patients had epileptic seizures and 3 had refractory epilepsy, in which the general effect of antiepileptic drugs was poor. Two patients developed central hypoventilation and received ventilator-assisted ventilation, which can be easily misdiagnosed as viral encephalitis because of similar clinical manifestations.

Dalmau et al. have reported that 72 patients were eventually diagnosed with anti-NMDAR encephalitis after the misdiagnosis of viral encephalitis because of similar clinical features [8]. Therefore, it is suggested that patients with combined psychiatric symptoms and seizures (especially recurrent refractory epilepsy) should be promptly screened for AE antibody series to avoid delay in the diagnosis and treatment. Several studies have found that the CSF of the patients with anti-NMDAR encephalitis showed a higher level of visible lymphocytosis, protein content, and oligoclonal bands, but they were not specific. Additionally, the cerebral MRI can be normal, which could show a high signal in the medial temporal lobe of the FLAIR sequence or the frontal and parietal lobe. The observations of the CSF examination and imaging studies are consistent with the report. However, the CSF routine examination, oligoclonal band detection, MRI, and other changes in the impact of examination do not belong to the specific diagnosis of AE indicators.

In this study, there were 4 cases of anti-LGI1 encephalitis, and the patients with such encephalitis usually showed typical symptoms of borderline encephalitis, such as severe short-range memory deficits and psychiatric symptoms. A considerable number of patients had hyponatremia and rapid ocular sleep disorders [11]. More than half of the patients with anti-LGI1 encephalitis experienced hyponatremia usually in the early stage, and it is easy to correct, which may be related to the abnormal secretion of antidiuretic hormone [12]. Some patients experienced seizures, which are usually caused by multidrug-resistant refractory epilepsy and can be alleviated by immunosuppressive therapy [13]. Approximately 85% of patients underwent MRI scans of the medial temporal lobe and showed unilaterally or hyperboloidly high signals [14, 15]. In the CSF, there was an increase in the lymphocytes and protein content and oligoclonal bands were also observed.

In this study, out of the total 4 patients, there were 3 males and 1 female, and all of them had seizures but not intractable epilepsy; the brain MRI of 2 patients showed bilateral hippocampal abnormal signals, and the other case showed recurrence after 6 months, and the cranial MRI after recurrence showed abnormal signals of the hippocampus, long T1 signal, slightly longer T2 signal, and a slightly higher signal in the FLAIR sequence. The early manifestations seen in 3 patients were memory loss and hyponatremia during early treatment, which could be quickly corrected. In addition, no tumor was identified.

There are 3 cases of anti-GABAB receptor encephalitis in this study, which is rare and only accounts for 14% of AE [16]. The main manifestation of those 2 cases is borderline encephalitis including cognitive dysfunction, memory loss, behavioral changes, and epilepsy (epileptic seizures can occur) [17, 18]. The patients with anti-GABAB receptor encephalitis primarily involved the damage in the hippocampus and temporal lobe of the brain [19]. Up to 80% of patients had tumors, mainly small-cell lung cancer (SCLC) [19–21]. Tumor and immunosuppressive therapy are effective against GABAB receptor encephalitis. Immunosuppressive agents can better control seizures compared with antiepileptic drugs. The long-term prognosis depends largely on the presence of potential cancer, and patients with SCLC have very poor prognoses [18, 22].

In this study, 3 patients presented with seizures at the onset, and antiepileptic drugs were ineffective on them. One patient had suffered memory injury, unresponsiveness, nonsense, and other cognitive impairments and mental disorders, and 2 patients had central hypoventilation. Three cases were treated with gamma globulin combined with hormone therapy, which alleviated the condition, and no recurrence of seizures was observed. However, irritability and other psychiatric symptoms remained.

By the screening of one case, it was found that the space was occupied by the right upper lung, but it was not clear through pathology; the patient's family gave up the treatment and the patient died outside the hospital. The result of the other 2 patients showed no tumor. It is recommended to regularly review the observations even after discharge. It is recommended that lung CT should be routinely examined in patients with anti-GABAB receptor encephalitis, and a qualified hospital-completed positron emission tomography- (PET-) CT scan may be helpful in the detection of tumors that are negative for lung CT. According to the recommendations in the literature [13], patients with clinically diagnosed anti-GABAB receptor encephalitis should be followed up regularly for tumor markers, chest CT, and PET examinations, even if no tumor is found.

According to the “Expert Consensus on Diagnosis and Treatment of Autoimmune Encephalitis” published by the Chinese Journal of Neurology in February 2017, AE treatment includes immunotherapy, symptomatic treatment of seizures and psychiatric symptoms, supportive care, rehabilitation, and resection of the tumor along with other antitumor treatments [23]. Immunotherapy is divided into first-line immunotherapy (glucocorticoids, intravenous immunoglobulin (IVIg), and plasma exchange), second-line immunotherapy (rituximab and intravenous cyclophosphamide), and long-term immunotherapy (phenol esters, azathioprine, etc.).

First-line immunotherapy is the preferred treatment, and second-line immunosuppressive drugs are mainly used in patients with poor response to the first-line immunotherapy. The long-term immunotherapy drugs are used mainly for relapse cases and can also be used for first-line immunotherapy in patients with poor response and also in patients with antitumor anti-NMDAR brain inflammation. Immunotherapy drugs may be used as the first-line treatment for AE. In this study, 13 patients received immunotherapy, out of which 4 patients received only hormone therapy, and 9 patients received hormones combined with IVIg treatment, including 3 cases of ineffective first-time IVIg treatment where two IVIg treatments were conducted and the curative effect of treatment was obvious. Out of the 13 cases, the treatment was abandoned in 2 cases, one because of status epilepticus and the other because of lung cancer; in the other cases, it was controlled obviously in epilepsy and cognitive impairment and abnormal mental behavior were also significantly improved. In addition, there are 8 cases of suspected AE, although there were no positive results of the AE antibody test, and after the immunotherapy (hormone and IVIg), the disease was significantly eased. It is recommended that first-line immunotherapy (hormones and IVIg) is still preferred in patients with AE. The IVIg may be repeated every 2–4 weeks if they are ineffective, and immunosuppressants may be considered if the effect is still poor. Patients suspected of AE may be immunized despite a negative antibody test.

In summary, although the number of cases of this study is small, the clinical manifestations of AE have their uniqueness, which is enough to enhance our cognition about AE. It generally has an acute or subacute onset, and the clinical manifestations are mostly epilepsy, cognitive dysfunction, and mental disorders. The general auxiliary blood and CSF tests can help eliminate the infection and rheumatic diseases, and the imaging findings prompt encephalitis. The results of the EEG examination are mostly abnormal but with no specific diagnostic significance. The diagnosis of the disease depends mainly on a detailed history, clinical manifestations, and positive results for specific NSAbs tests. The treatment is mainly done by immunosuppressive therapy. Patients with AE also need an early diagnosis, which could help avoid missed or misdiagnosis and initiate the immunosuppressive therapy as soon as possible, which can effectively reduce mortality and morbidity.

## Figures and Tables

**Table 1:** The clinical data of 13 patients with autoimmune encephalitis.

	1	2	3	4	5	6	7	8	9	10	11	12	13
Age (years)	56	60	46	46	15	24	30	68	61	54	56	17	59
Sex	Female	Male	Male	Male	Male	Male	Female	Male	Male	Male	Female	Male	Female
Initial symptoms	Psychological symptoms	Epileptic seizure	Epileptic seizure	Advanced dementia	Epileptic seizure	Epileptic seizure	Psychological symptoms	Epileptic seizure	Epileptic seizure	Epileptic seizure	Psychological symptoms	Psychological symptoms	Psychological symptoms
Psychological and behavioral abnormalities	+	-	+	+	+	-	+	+	+	+	+	+	+
Epileptic seizure	+	+	+	+	+	+	+	+	+	+	+	+	+
Cognition impairment	+	+	+	+	-	+	+	+	+	+	+	+	+
Mechanical ventilation	-	-	+	-	+	-	+	-	+	-	-	-	-
MRI	-	-	-	+	-	-	-	+	-	+	-	+	-
Cancer screening	-	-	Lung cancer	-	-	-	-	-	-	-	-	-	-
Treatment	Glucocorticoid	Glucocorticoid	Glucocorticoid, IVIg	Glucocorticoid	Glucocorticoid, IVIg	Glucocorticoid, IVIg(2)	Glucocorticoid, IVIg	Glucocorticoid, IVIg	Glucocorticoid, IVIg (2)	Glucocorticoid, IVIg	Glucocorticoid, IVIg	Glucocorticoid, IVIg	Glucocorticoid, IVIg
Outcome	Improved	Improved	Abandoned	Improved	Improved	Improved	Abandoned	Improved	Improved	Improved	Improved	Improved	Improved
AE antibody testing	Anti-NMDA receptor	Anti-LG11 antibody	Anti-GABAB receptor	Anti-LG11 antibody	Anti-NMDA receptor	Anti-NMDA receptor	Anti-NMDA receptor	Anti-GABAB receptor	Anti-GABAB receptor	Anti-LG11 antibody	Anti-LG11 antibody	Anti-NMDA receptor	Anti-NMDA receptor

Abbreviations: AE: autoimmune encephalitis; NMDA: N-methyl-D-aspartate; GABAB: *γ*-aminobutyric acid B; LGl1: leucine-rich glioma 1; IVIg: intravenous immunoglobulin; MRI: magnetic resonance imaging.

## Data Availability

The data used to support the findings of this study are available from the corresponding author upon request.
